# Novel Treatment for Mitigating Condensate Bank Using a Newly Synthesized Gemini Surfactant

**DOI:** 10.3390/molecules25133030

**Published:** 2020-07-02

**Authors:** Amjed Hassan, Mohamed Mahmoud, Muhammad Shahzad Kamal, Syed Muhammad Shakil Hussain, Shirish Patil

**Affiliations:** 1 Department of Petroleum Engineering, King Fahd University of Petroleum & Minerals, Dhahran 31261, Saudi Arabia; g201205100@kfupm.edu.sa; 2 Center for Integrative Petroleum Research, King Fahd University of Petroleum & Minerals, Dhahran 31261, Saudi Arabia; shahzadmalik@kfupm.edu.sa (M.S.K.); smshakil@kfupm.edu.sa (S.M.S.H.)

**Keywords:** condensate bank, novel treatment, gemini surfactant, wettability alteration, capillary forces

## Abstract

Condensate accumulation in the vicinity of the gas well is known to curtail hydrocarbon production by up to 80%. Numerous approaches are being employed to mitigate condensate damage and improve gas productivity. Chemical treatment, gas recycling, and hydraulic fracturing are the most effective techniques for combatting the condensate bank. However, the gas injection technique showed temporary condensate recovery and limited improvement in gas productivity. Hydraulic fracturing is considered to be an expensive approach for treating condensate banking problems. In this study, a newly synthesized gemini surfactant (GS) was developed to prevent the formation of condensate blockage in the gas condensate reservoirs. Flushing the near-wellbore area with GS will change the rock wettability and thereby reduce the capillary forces holding the condensate due to the strong adsorption capacity of GS on the rock surface. In this study, several measurements were conducted to assess the performance of GS in mitigating the condensate bank including coreflood, relative permeability, phase behavior, and nuclear magnetic resonance (NMR) measurements. The results show that GS can reduce the capillary pressure by as much as 40%, increase the condensate mobility by more than 80%, and thereby mitigate the condensate bank by up to 84%. Phase behavior measurements indicate that adding GS to the oil–brine system could not induce any emulsions at different salinity levels. Moreover, NMR and permeability measurements reveal that the gemini surfactant has no effect on the pore system and no changes were observed in the T_2_ relaxation profiles with and without the GS injection. Ultimately, this work introduces a novel and effective treatment for mitigating the condensate bank. The new treatment showed an attractive performance in reducing liquid saturation and increasing the condensate relative permeability.

## 1. Introduction

**Figure 1:**
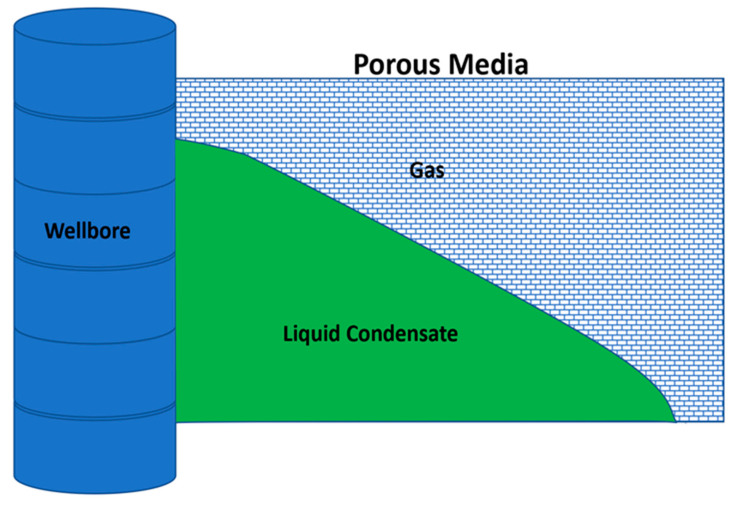
Effect of condensate bank on gas production.

Several problems may be encountered during hydrocarbon production from gas condensate reservoirs [1]. One of the main problems is the development of condensate bank around the production well [2,3,4]. During production, when the reservoir pressure drops below the dew point pressure, heavy hydrocarbons may condensate and precipitate around the borehole [1,4,5,6]. The accumulation of condensate liquid poses a serious formation damage problem and thus may reduce gas productivity significantly [7,8,9]. The formation damage becomes more serious in tight reservoirs, where the condensate bank becomes immobile and blocks the gas flow [3,10,11]. Figure 1 illustrates a schematic diagram of the condensate bank in the near-wellbore region. The accumulated liquid restricts the gas flow and thus reduces gas production.

Several methods are employed to alleviate the condensate damage and improve reservoir productivity [2,3,4]. The most common treatments are gas recycling, chemical injection, and hydraulic fracturing. Gas recycling is used to increase and maintain the formation pressure above the dew point curve in the near-wellbore region [12,13], thereby re-vaporizing the condensate liquid into the gas phase [14,15]. Different gases are used in recycling treatment, such as methane, nitrogen, and carbon dioxide [2,16]. However, the main limitation of gas recycling is that the treatment needs to be repeated every 6 to 9 months in order to maintain its effectiveness [2,3,4,14,16]. Besides the logistical challenges associated with gas transportation, there exists an additional challenge of on-site gas handling during the gas recycling treatment [3,4,17]. Alternatively, hydraulic fracturing is used to mitigate the condensate bank by inducing conductive channels between the wellbore and the reservoir matrix [18,19,20]. The created fractures improve the flow paths available and decrease the drawdown pressure [20,21,22]. However, after several months of production, the condensate bank may develop around the wellbore due to the reduction in reservoir pressure [3,4].

Chemical treatments provide an encouraging solution for alleviating the condensate damage [3,4,23,24,25,26]. Chemical solutions are used to dissolve the condensate liquid or to change the rock wettability [27,28,29]. The solvent injection is used to decrease the interfacial tension at the gas/condensate interface and hence improve the condensate mobility [30,31]. Wettability alteration chemicals are injected to change the rock wettability to less liquid-wet state, thereby preventing the liquid accumulation [10,23,28,32]. Extensive laboratory experiments and numerical modeling have been conducted to evaluate the efficiency of wettability alteration treatments on mitigating the condensate bank [7,11,23,26,28,33,34,35,36]. Coreflooding, contact angle, and spontaneous imbibition experiments are the common methods used to investigate the wettability alteration during condensate removal treatments [23,37,38,39,40].

Al-Anazi et al. [23] examined the performance of fluorochemical and silane fluids in preventing the condensate bank for sandstone and carbonate formations. They studied the impacts of chemical type, injected volume, and rock properties on the condensate removal efficiency. They observed that the gas relative permeability can be increased by up to 42% and 17% for sandstone and carbonate rocks, respectively, using the wettability alteration mechanism. Li et al. [41] used fluorocarbon surfactant to improve the condensate removal by changing the wettability conditions to that of preferential gas wet. They reported that the chemical used was stable at temperature up to 170 °C and can be applied under high-salinity conditions up to 70,000 ppm. They further concluded that liquid removal of 76.5% could be achieved using the fluorocarbon surfactant. Zheng and Rao [39] reported that anionic surfactants can change the wettability condition from preferential liquid-wet to weak liquid-wet state. The chemicals used were able to reduce the interfacial tension at the liquid/gas interface and thereby improved the gas relative permeability.

Al-Yami et al. [25] reported a successful field treatment for removing the condensate bank using a polymeric fluorinated surfactant. The chemical treatment increased the condensate production from around 280 bbl/D to 1150 bbl/D; however, the gas production increased by around 14% compared to the initial gas production. Additionally, the production profiles indicated that the liquid saturation was very high after the treatment, and the removal of condensate bank was temporary. Therefore, they concluded that the injection of polymeric fluorinated surfactants is not an effective approach for mitigating the condensate bank. Sharifzadeh et al. [30] used fluorinated silica to alter the wettability condition from preferential liquid-wet to intermediate gas-wet. Ethanol and nonafluorohexyltriethoxy silane were also injected with the surfactant fluid. Different measurements were conducted to examine the chemical performance including contact angle measurements, coreflooding tests, and thermogravimetric experiments. They reported that gas production increased by approximately 67% after the treatment. Moreover, Karandish et al. [31] applied anionic fluorinated treatment for altering the carbonate rock wettability for the gas condensate reservoir. Different mixtures of solvents and surfactants were used to change the rock wettability from water-wet to intermediate gas-wet, thereby enhancing the gas relative permeability. They reported that the chemicals used increased the gas relative permeability by 70%, at reservoir conditions.

Recently, nanoparticles were used to alter the rock wettability for both carbonate and sandstone formations [37,42,43,44,45,46]. Aminnaji et al. [42] and Erfani Gahrooei et al. [43] found that nanofluid can considerably change the wettability of carbonate and sandstone rocks to preferentially gas wet by aging the rocks for around one day. They mentioned that the gas wetting surface could be achieved by increasing the surface roughness and decreasing surface free energy. Sayed et al. [44,45] studied the performance of nanoparticles-based treatment in mitigating the condensate and water bank for sandstone reservoirs. They used fluorinated silica nanoparticles (NP) to adjust the wettability condition from liquid wet to neutral or intermediate gas wet. The nanoparticles used had an average size between 135 to 400 nm, and the NP concentration was around 0.065 *wt*%. They reported that the nanoparticle-based treatment was able to alter the rock wettability toward strongly non-liquid wetting and thereby improved the gas relative permeability by around 37%. However, they concluded that the NP sizes should be optimized based on the pore sizes of the treated formations to avoid pore-throat plugging. Furthermore, Safaei et al. [37] investigated the wettability alteration of carbonate reservoirs using Fe_3_O_4_ nanoparticles coated with hydroxyapatite (NPs-HAp) or polyvinyl alcohol (NPs-PVA). The nanoparticles used in this study had an average size of 26.3 nm, and the NP concentrations varied between 0.4 and 0.5 *wt*%. They reported that the nanoparticles coated with HAp significantly changed the rock wettability from oil-wet to gas-wet state. Additionally, they reported that the NP-based treatment improved the gas rate by around 63.5% and reduced the pressure drop by 33% due to the wettability alteration to preferentially gas-wet state.

The common condensate removal methods, such as gas recycling and hydraulic fracturing, are expensive treatments. Given the current volatility of extremely low oil prices, the application of these treatments will not be economically beneficial. Recently, nanoparticle-based treatment has been presented as a novel technique for changing the wettability condition. However, in tight gas reservoirs, nanoparticle injection may increase the possibility of pore-throat plugging, which will impair the reservoir permeability. Alternatively, chemical treatments present cost-effective, low-risk, and long-term solutions. Chemical injection showed an effective performance in altering the rock wettability and thereby enhancing gas production. However, under high-temperature and -salinity conditions, the injected chemicals may hydrolyze and lose their efficiency [24].

This paper introduces an innovative condensate treatment using a newly synthesized gemini surfactant (GS). The used chemical exhibits an attractive performance profile and able to combat condensate banking related challenges. In this treatment, the newly synthesized GS can be injected into the tight formation to prevent the accumulation of condensate liquid around the wellbore. Flushing the near-wellbore area with GS will change the rock wettability to less oil-wet conditions and therefore enhance the condensate mobility. GS can be adsorbed on the rock surface in the form of a very thin film. The adsorbed GS layer is stable and can be sustained for a long time; hence, the presented treatment using GS can be considered as a long-term solution for condensate banking issues. Additionally, the injected chemical can decrease the capillary pressure holding the condensate and thereby improve the condensate removal efficiency. In this study, coreflood, relative permeability, NMR, and phase behavior measurements were carried out. The impacts of GS treatment on the condensate removal, relative permeability, capillary pressure, fluid mobility, and rock porosity system were investigated.

## 2. Results and Discussion

In gas condensate reservoirs, the gas flow into the wellbore can be blocked due to the accumulation of condensate liquid around the production well. However, the injection of surfactant fluids can adjust the rock wettability and thus can help in preventing the liquid accumulation. This work presents the performance of GS in alleviating the condensate bank and improving gas productivity. First, the condensate recovery due to the injection of GS into tight rock samples is discussed. Next, the improvement in condensate relative permeability after the treatment is analyzed using JBN (Johnson, Bossler, and Neumann) method [47]. Additionally, the reduction in capillary pressure and the improvement in fluid mobility due to the GS injection are determined. Finally, the impact of GS treatment on the rock porosity system is examined. Results of coreflood, relative permeability, phase behavior, and NMR measurements are thoroughly discussed.

### 2.1. Condensate Removal

**Figure 2:**
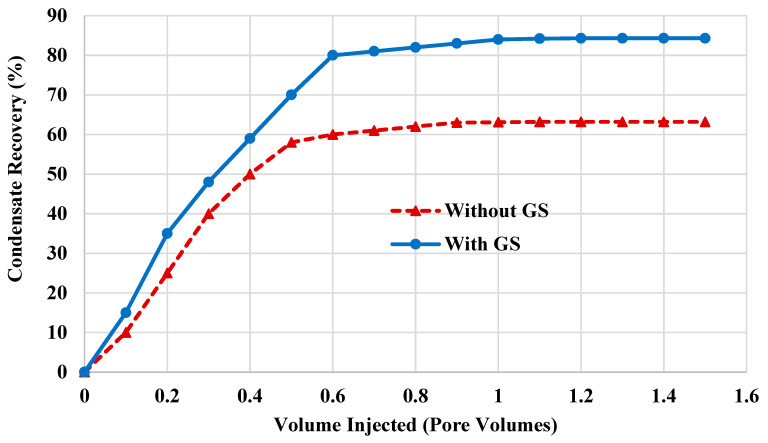
Condensate recovery using gemini surfactant (GS) compared to saline brine injection.

Figure 2 shows the condensate recovery from 6-inch Scioto sandstone with and without flushing with GS. This rock sample has an average porosity of 13%, and the absolute permeability is 0.27 mD. Two flooding cases were investigated, and the KCl solution (3 *wt*%) was injected through the core to recover the condensate in both cases. In this work, the oil-brine system was used to minimize the uncertainty associated with condensate re-vaporization, and to focus on the condensate removal using wettability alteration induced by the newly synthesized gemini surfactant. In the first experiment, the core was saturated using KCl brine and then the condensate bank situations were represented by saturating the rock sample with oil. After that, KCl brine was injected again to recover the condensate. However, in the second experiment, a 0.5 *wt*% GS solution was injected to flush the core sample before saturating the rock sample with condensate. Thereafter, KCl brine was injected to remove the condensate liquid from the tight rock. The main difference between the two experiments is that GS was used in the second experiment, while all other parameters were kept unchanged. All injection experiments were carried out under high-pressure (1500 psi) and high-temperature (100 °C) conditions. In the first case, where no GS was used, the condensate recovery was 63.2%. While in the second case, where GS was injected, around 84% of the hydrocarbon liquid was recovered. Therefore, the addition of GS to the rock-fluid system led to an increase of 20% in the condensate recovery. The increase in condensate recovery can be attributed to the fact that the injected chemicals can cover the pore surface and enhance the water wetness of the rock surface, thereby facilitating the condensate mobility compared to the case without using GS. The wettability alteration induced by GS was confirmed by relative permeability measurements, and capillary pressure and fluid mobility calculations, as will be discussed in the next sections of this paper. Consequently, in field applications, GS can be used as a completion fluid to adjust the wettability condition around the wellbore and thus prevent the accumulation of condensate liquid.

### 2.2. Wettability Alteration

**Figure 3:**
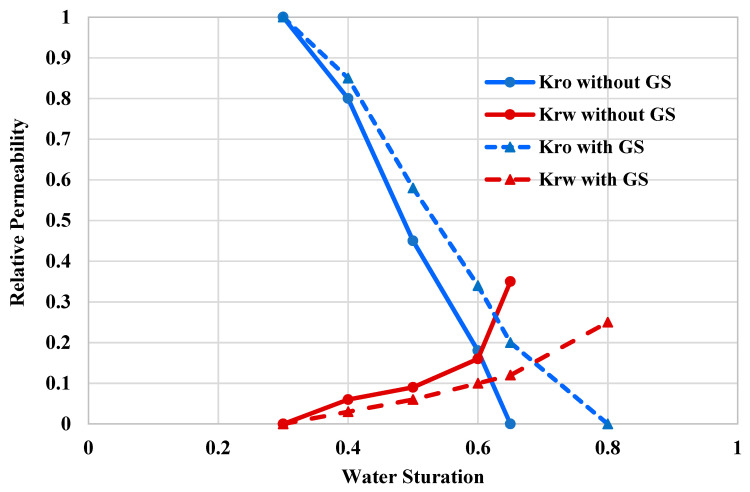
Relative permeability curves, with and without GS.

Injection of GS into the near-wellbore region can alter the rock wettability of this region to less oil-wet state. Several researchers reported the efficacy of gemini surfactants in altering the rock wettability [48,49,50]. In addition, gemini surfactant possesses other interesting properties, such as low critical micelle concentration (CMC) [51,52]. In this work, GS was used to reduce the oil-wetness and therefore enhance the condensate mobility. The wettability alteration was evaluated by measuring the relative permeability with and without introducing the GS into the rock-fluid system. Figure 3 shows the oil and water relative permeability curves, with and without GS. The rock sample is water wet because the intersection point between the oil and water relative permeability is 0.6, which is more than 0.5 [53]. Additionally, the relative permeability of the water phase at the residual oil saturation is 0.35, which indicates water wetting conditions as well. However, flushing the rock with GS solution increased the water wetness of the core sample and shifted the intersection point to the right from 0.6 to 0.68 and reduced the water relative permeability at the residual oil saturation from 0.35 to 0.24. Therefore, the oil mobility will be enhanced compared to the other case, where GS was not used. At a water saturation of 0.6, the relative permeability of the condensate increased from 0.17 to 0.34 after flushing the core with GS. The condensate relative permeability almost doubled at this fluid saturation. The surfactant was adsorbed on the rock surface in the form of very thin film and thus prevented the accumulation of condensate liquid. However, the injected chemical also increased the water wetness, which is not desirable in the case of high water saturations. It should be noted that GS treatment increases the water-wetness only when the water saturation is higher than 0.6, which is not the common case in most gas reservoirs. Moreover, after the treatment, the residual oil saturation was reduced by around 43%, while no considerable changes were observed in the critical water saturation, indicating that GS treatment can be implemented safely when the water saturation is less than 0.6. Ultimately, injection of GS into the tight rock can lead to considerable alterations in rock wettability and thereby prevent the condensate accumulation; however, precautions should be taken in the case of high-water saturation (more than 0.6) to avoid the creation of water bank.

**Figure 4:**
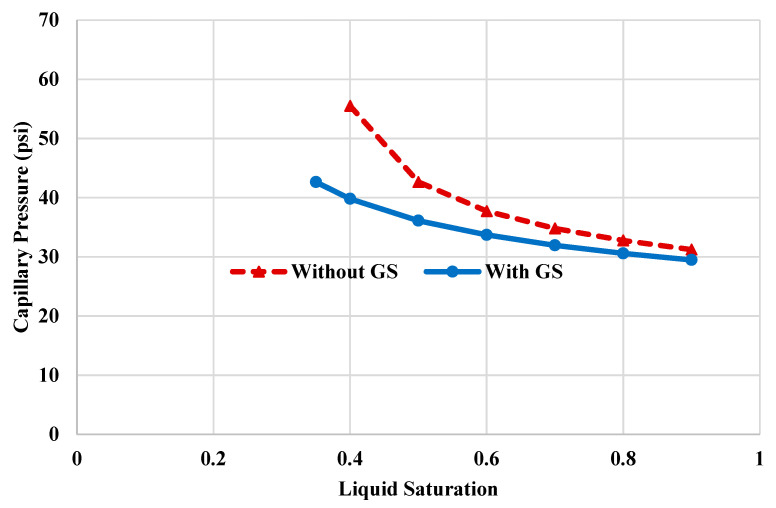
Profiles of capillary pressure with and without injection of GS.

**Figure 5:**
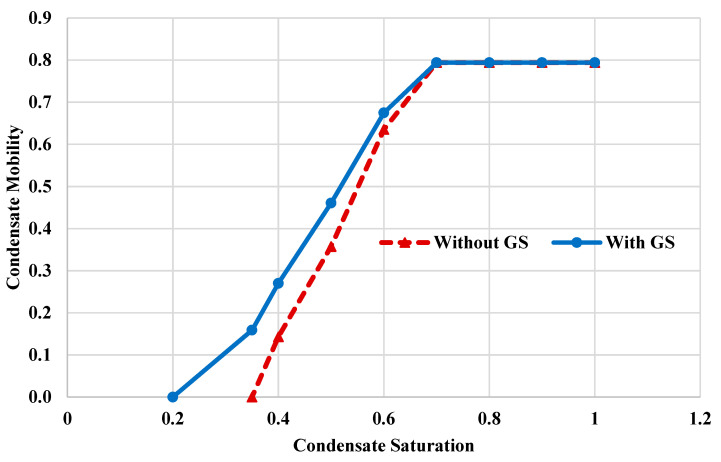
Profiles of condensate mobility with and without GS injection.

The alteration of rock wettability, due to GS injection, can significantly reduce the capillary pressure (Pc). Figure 4 shows the capillary pressure curves with and without the injection of GS. Equations (1) and (2) [53,54] were used to calculate the capillary profiles as a function of the fluid saturation. The surfactant injection led to a reduction in the capillary pressure by around 40%. At liquid saturation of 0.4, the Pc was reduced from 55.5 psi to around 39.8 psi due to the GS injection. Additionally, the surfactant treatment was able to reduce the condensate saturation by more than 30%, at the same capillary forces. Therefore, GS treatment can enhance the condensate mobility and minimize liquid accumulation. Furthermore, the relative permeability curves were used to estimate the condensate mobility. Figure 5 shows the effective condensate mobility profiles, with and without GS injection. The condensate mobility was determined using Equation (3). At high liquid saturation (above 0.6), the condensate mobility is similar both cases, which could be attributed to the fact that the condensate is present at the center of the pore space and the rock wettability condition has a minor impact on the condensate flow. However, when the fluid saturation is smaller than 0.4, the condensate mobility increased by up to 85% due to the injection of GS. At this saturation, most of the condensate liquid is precipitated on the rock surface, confirming that the condensate mobility strongly depends on the surface wettability. Injection of GS will alter the rock surface toward less oil-wet conditions and therefore improve the condensate mobility.

(1) $$P_{c}=P_{d}S_{e}{}^{-\frac{1}{\lambda }}$$

(2) $$S_{e}=\frac{S_{\mathrm{con}}-S_{\mathrm{conr}}}{1-S_{\mathrm{conr}}}$$

(3) $$M=\frac{{\mathrm{kk}}_{r}}{\mu }$$

where P_c_ is the capillary pressure (psi), P_d_ is the threshold or displacement pressure (psi), S_con_ is the condensate saturation, S_conr_ is the residual condensate saturation, and λ is the pore size factor, λ is 4.17 for sandstone rocks. In Equation (3), M indicates the fluid mobility, k is the rock permeability (mD), k_r_ is the relative permeability, and μ is the fluid viscosity (cP).

### 2.3. Phase Behavior

**Figure 6:**
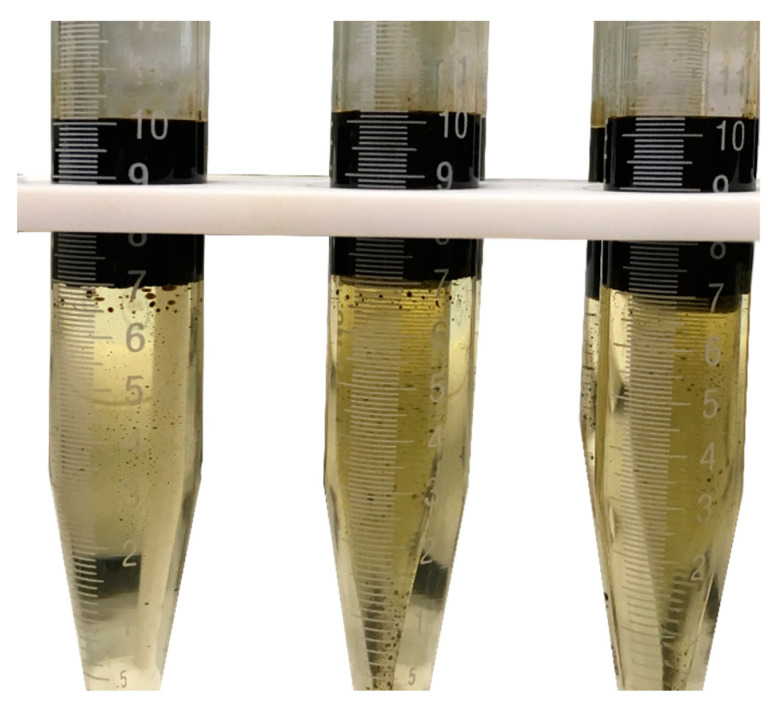
Oil and brine system after adding GS at different salinity levels.

Chemical injection into the near-wellbore region can lead to significant changes in the behavior of the fluid in this region. The injected chemicals can positively impact the near-wellbore region by altering the rock wettability toward less-oil wet conditions. However, it can also reduce the hydrocarbon flow into the wellbore by inducing stable emulsions around the production well. The chemical injection may lead to creating water-in-oil or oil-in-water emulsions due to the disturbance of formation fluids around the wellbore. The formation of emulsion bank in the near-wellbore region can induce formation damage similar to the condensate banking problems. Therefore, the impact of GS on oil behavior was evaluated by monitoring the oil-surfactant system at different salinity levels. Figure 6 shows images of oil-GS samples with different brine salinity, at ambient conditions. As can be seen from these images, no emulsion was induced at any of the salinity levels. This observation could indicate that the injection of GS into the condensate bank region will not form an emulsion blockage. Additionally, the results of coreflood experiments, conducted in this study, confirmed that no emulsion damage was induced. In the coreflood experiments conducted in this work, more condensate liquid was recovered for the rock sample treated with GS compared to the case where no GS was used. Additionally, the effluent of coreflooding does not have any emulsion.

### 2.4. Pore Size Characterizations

**Figure 7:**
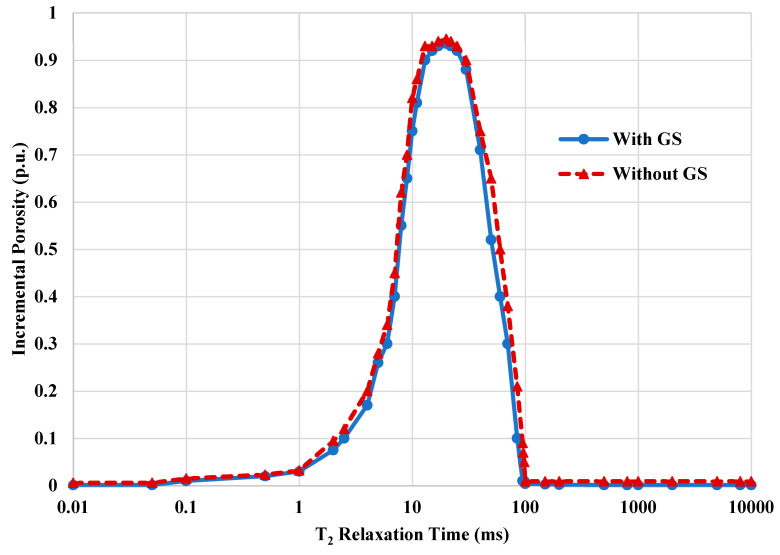
Porosity profiles for the rock sample with and without GS treatment.

Flushing the rock sample with GS led to mobilization of the condensate liquid and enhancement of its relative permeability. However, the injected chemicals showed minor changes in the rock pore network. In this work, nuclear magnetic resonance (NMR) was used to study the pore network using the T_2_ relaxation time. Figure 7 shows the T_2_ profiles for the rock sample treated with and without GS. The changes in the rock porosity system due to a certain treatment can be captured by studying the behavior of T_2_ profiles before and after the treatment. In general, a shift in the relaxation time into the right side (higher relaxation times) indicates an improvement in the rock porosity system. If the T_2_ profile is shifted into the left side (lower relaxation times), it indicates damage has been inducted into the treated rock. In this work, there are no considerable changes in the T_2_ profiles, indicating that the injected chemical did not dissolve the rock surface or create new pore space, also, no damage was induced due to the GS injection. The surfactant was adsorbed on the rock surface in the form of a very thin film, making the rock surface water wet without affecting the rock permeability and porosity. This confirmed that the newly synthesized surfactants are compatible with the sandstone rocks and adsorbed on the rock surface at the micro-level because no changes to the permeability or porosity were observed. The permeability measurements confirmed that injection of GS chemicals into tight rock sample did not affect the rock permeability. The core permeability with and without GS was the same, 0.27 mD, and the core porosity with and without GS was 13%.

## 3. Materials and Methods

### 3.1. Rocks and Fluids

In this study, Scioto sandstone rock samples with mineralogical composition provided in Table 1 were used. The samples showed a high percentage of quartz (71%) and illite (18%), while small percentages of chlorite and feldspars were observed. Permeability and porosity measurements indicate that the rocks samples were tight; the average rock porosity and permeability were 12.8% and 0.26 mD, respectively. The petrophysical properties are listed in Table 2.

**Figure 8:**
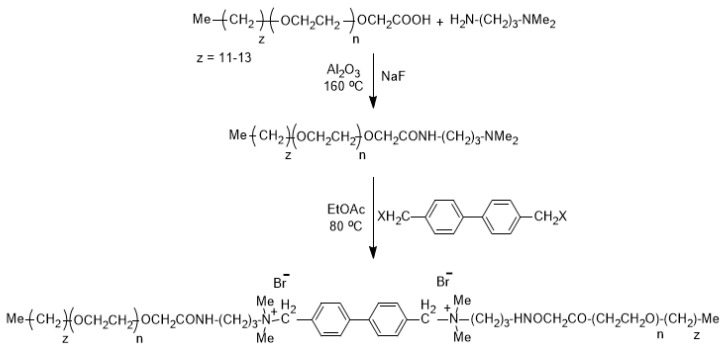
Synthetic protocol of cationic gemini surfactant (GS) used in this work.

Additionally, the condensate liquid of 45° API (American Petroleum Institute) was used. The condensate liquid was used to saturate all rock samples at high pressure conditions to ensure representative results. Moreover, the synthesized GS was used. The synthetic outline and the chemical structure of the surfactant are illustrated in Figure 8. The detailed description of the experiments and spectral data analysis of the GS is presented in our preceding article [55]. The surfactants were synthesized by the reaction of the acid with the amine in a round-bottom flask at 160 °C for 6 h. Sodium fluoride was used as a catalyst. After 6 h, additional amine was added to achieve complete conversion of acid. The intermediate amide was finally reacted with 4,4′-bis(bromomethyl)biphenyl to achieve the final product. The critical micelle concentration of the surfactant is 0.00887 mmol/L at 30 °C. A 3 *wt*% KCl brine was used to measure the absolute rock permeability and to prepare the surfactant solutions at the desired chemical concentrations.

### 3.2. Experiments

**Figure 9:**
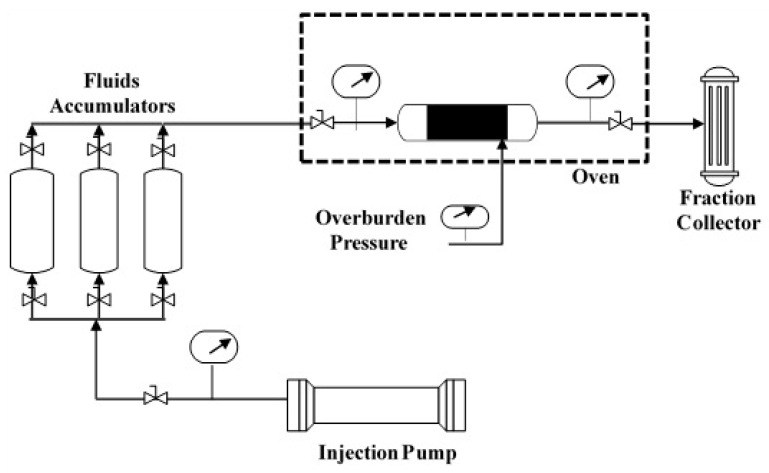
A schematic diagram of the coreflood setup used in this work.

In this work, several experiments were conducted, including coreflood, relative permeability, NMR, and phase behavior measurements. The coreflooding experiments were performed by injecting the GS into the tight rocks at high pressure conditions. Backpressure of 500 psi, and overburden pressure of 1500 psi was applied. Additionally, high-temperature conditions were implemented and a system temperature of 100 °C was used. Figure 9 shows a schematic diagram of the coreflooding system used in this study. The experimental setup consists of injection pumps, pressure regulators, oven, and data acquisition systems. Moreover, the coreflooding system was used to measure the relative permeabilities for water and condensate using the unsteady-state method. Two relative permeability measurements were conducted, with and without the GS. First, the rock sample was saturated with KCl brine at high pressure, then, the KCl brine was displaced and the rock sample was saturated with condensate liquid. The volumes of produced brine and oil were recorded with time, until no more brine was produced. In the second experiment, 0.5 *wt*% GS was used to saturate the rock sample, applying similar experimental conditions. The GS was then displaced by injecting the condensate oil at a constant flow rate. The oil and water relative permeabilities were determined using the JBN method [47].

In addition, phase behavior measurements were carried out by evaluating the impacts of GS on the oil phase behavior, using different brine salinity (0 to 2M NaCl). All phase behavior experiments were performed at an ambient temperature of 23 °C. Finally, NMR measurements were conducted using the rock sample treated with and without GS injection. Core Analyzer Oxford Instrument was used to measure the T_2_ relaxation time. The rock samples were prepared for the NMR measurements by cleaning the samples using the Soxhlet method, and then the rock samples were saturated using 3 *wt*% KCl brine. The NMR measurements were conducted using the same experimental conditions, to minimize the NMR uncertainty.

## 4. Conclusions

This work introduces a novel treatment for mitigating the condensate bank in tight gas condensate reservoirs using a newly synthesized GS. Several measurements were used to assess the performance of the proposed treatment including coreflood, relative permeability, phase behavior, and NMR measurements. Based on this work, the following conclusions can be drawn;

- Flushing the tight rock with a gemini surfactant can remove more than 84% of the condensate liquid.
- Injection of GS can decrease the capillary forces by 40% and increase the condensate mobility by more than 80%.
- Injection of GS into the condensate region will not induce any emulsions, at different salinity levels.
- GS does not affect the pore system; no changes were observed in the T_2_ relaxation profiles with and without the GS injection.
- Finally, the new treatment showed an attractive performance in reducing liquid saturation and increasing the condensate relative permeability.

## Figures and Tables

**Table 1:** Mineralogical compositions of the rock samples.

Minerals	Chemical Formula	*wt*%
Calcium Feldspar	CaAl_2_Si_2_O_8_	5
Chlorite	(Fe, Mg)_5_Al(Si_3_Al)O_10_(OH)_8_	4
Illite	K_0.65_Al_2_[Al_0.65_Si_3.35_O_10_](OH)_2_	18
Potassium Feldspar	KAlSi_3_O_8_	2
Quartz	SiO_2_	71
Total		100

**Table 2:** Petrophysical properties of the rock samples.

Sample Index	Length (in)	Diameter (in)	Pore Volume (ml)	Porosity (%)	Permeability (mD)
Core 1	6	1.5	22.59	13	0.27
Core 2	6	1.5	21.72	12.5	0.24
